# Androgen receptor splicing variant 7 (ARV7) inhibits docetaxel sensitivity by inactivating the spindle assembly checkpoint

**DOI:** 10.1016/j.jbc.2021.100276

**Published:** 2021-01-09

**Authors:** Bingbing Yu, Yanan Liu, Haoge Luo, Jiaying Fu, Yang Li, Chen Shao

**Affiliations:** Department of Pathophysiology, College of Basic Medical Sciences, Jilin University, Changchun, China

**Keywords:** prostate cancer, ARV7, docetaxel, spindle assembly checkpoint, mitotic slippage, UBE2C, ADT, androgen-deprivation therapy, APC/C, anaphase-promoting complex/cyclosome, AR, androgen receptor, AR-FL, androgen receptor (full-length), ARV7, androgen receptor splicing variant 7, BubR1, mitotic checkpoint serine/threonine-protein kinase BUB1 beta, CDC20, cell division cycle protein 20, CRPC, castration-resistant prostate cancer, DTX, docetaxel, FACS, fluorescence-activated cell sorting, LBD, ligand-binding domain, Mad2, mitotic arrest deficient 2, MCC, mitotic checkpoint complex, OE, overexpression, PCa, prostate cancer, PSA, prostate-specific antigen, SAC, spindle assembly checkpoint, SASP, senescence-associated secretary phenotype, UBE2C, ubiquitin-conjugating enzymes 2C

## Abstract

The clinical efficacy of docetaxel (DTX) in prostate cancer treatment is barely satisfactory due to diverse responses of the patients, including the development of resistance. Recently, aberrant androgen receptor (AR) signaling, including expression of the constitutively active ARV7, was reported to contribute to DTX resistance. However, the underlying molecular mechanism remains largely unknown. Of note, previous studies have highlighted that ARV7, unlike its parental AR, potentially favors the expression of some genes involved in cell cycle progression. Since DTX mainly targets microtubule dynamics and mitosis, we wanted to test whether ARV7 plays a specific role in mitotic regulation and whether this activity is involved in DTX resistance. In the present study, we found that ARV7 mediates DTX sensitivity through inactivating the spindle assembly checkpoint (SAC) and promoting mitotic slippage. By shifting the balance to the slippage pathway, ARV7-expressing cells are more likely to escape from mitotic death induced by acute DTX treatment. Furthermore, we also identified E2 enzyme UBE2C as the primary downstream effector of ARV7 in promoting the SAC inactivation and premature degradation of cyclin B1. Moreover, we showed that combination treatment of DTX and an inhibitor of mitotic exit can exert synergistic effect in high ARV7-expressing prostate cancer cells. In sum, our work identified a novel role of ARV7 in promoting DTX resistance and offering a potential path to combat DTX resistance related to abnormal activation of the AR signaling and mitotic dysregulation.

Despite considerable effort has been made in recent years, prostate cancer (PCa) remains to be one of the most common causes of cancer-dependent deaths in men worldwide (1). Since PCa cells rely on androgen receptor (AR) signaling for growth and survival, androgen-deprivation therapy (ADT) is considered as the major treatment option for PCa patients. However, although most of the patients initially respond to ADT well, castration-resistant prostate cancer (CRPC) eventually occurs after several years and then progresses to metastatic diseases, leading to the treatment failure (2). Notably, most CRPC cells are still dependent on active AR signaling for survival, which is primarily due to aberrant alterations of the AR pathway including gain-of-function mutations, overexpression of the AR gene, stimulation by other signaling pathways as well as expression of constitutively active AR-splicing variants (3).

Recently, expression of AR-splicing variants (ARVs) emerges as one crucial mechanism accounts for abnormal activation of the AR pathway (4). In general, ARVs are characterized as shortened forms of AR, which lack the C-terminal ligand-binding domain (LBD). Among all the identified variants, androgen receptor splicing variant 7 (ARV7) draws the most attention as it is the best studied ARV so far and also determined to be the most abundant form in cell lines, xenografts, and clinical samples (5). Accumulating studies have indicated that ARV7, no matter whether androgen is available, can constitutively localize to the nucleus and play a crucial role in PCa progression (6). ARV7 is rarely detectable in primary PCa, but its level is dramatically increased in response to ADT (7). In one recently conducted sample analysis, ARV7 was found to present in over 75% of patients who received ADT and correlate with worse PSA responses and overall survival (8). It is well known that ARV7 is capable of activating canonical AR downstream targets, mediating androgen-independent cell growth and resistance to AR-targeting therapy. Nevertheless, many studies have highlighted that ARV7 might also control a transcriptional program that is largely different from the one regulated by its parental AR (9, 10, 11). Specifically, expression of ARV7, but not AR, was positively correlated with the level of some cell cycle genes such as ubiquitin-conjugating enzymes 2C (*UBE2C*), raising the question that what are the roles of these unique regulations in PCa progression (12).

Docetaxel (DTX) has been the first-line drug for treating metastatic CRPC patients since 2004 although its effect is significantly impaired by the acquired drug resistance. Mechanistically, DTX can inhibit microtubule depolymerization, consequently activating the spindle assembly checkpoint (SAC) to arrest cells in mitosis and triggering apoptosis (13). Unattached kinetochores lead to the formation of the mitotic checkpoint complex (MCC) and suppression of Cdc20, rendering it unable to activate the anaphase-promoting complex/cyclosome (APC/C). Thus, as the E3 ligase for both securin and cyclin B1, the APC/C is able to control the sister-chromatid separation and mitotic exit (14, 15, 16). However, some cancer cells can prematurely degrade cyclin B1 before reaching the apoptotic signal intensity required for initiating mitotic cell death. By doing so, cells are able to exit mitosis without proper chromosome segregation and cytokinesis, through a process defined as mitotic slippage (17, 18). It is postulated that mitotic slippage can facilitate cancer cells to escape from taxol-induced cell death, leading to drug resistance (19, 20). Thus, deciphering how the SAC is precisely regulated is crucial for understanding the potential mechanism for DTX resistance. Importantly, previous studies have indicated that expression of ARV7 can promote DTX resistance in PCa (21, 22, 23). Of note, ARV7 was shown to utilize distinct pathways of nuclear import and also promote the nuclear localization of other ARVs during disease progression (24, 25). However, the molecular mechanism of how ARV7 exerts its function during controlling the fate of PCa cells under DTX treatment remains largely unexplored. In this study, we provided data to demonstrate that ARV7 promotes the survival of PCa cells under DTX treatment by inactivating the SAC and promoting mitotic slippage, offering a novel mechanism to explain how ARV7 contributes to DTX resistance.

## Results

### ARV7 plays oncogenic roles in PCa cells

**Figure 1:**
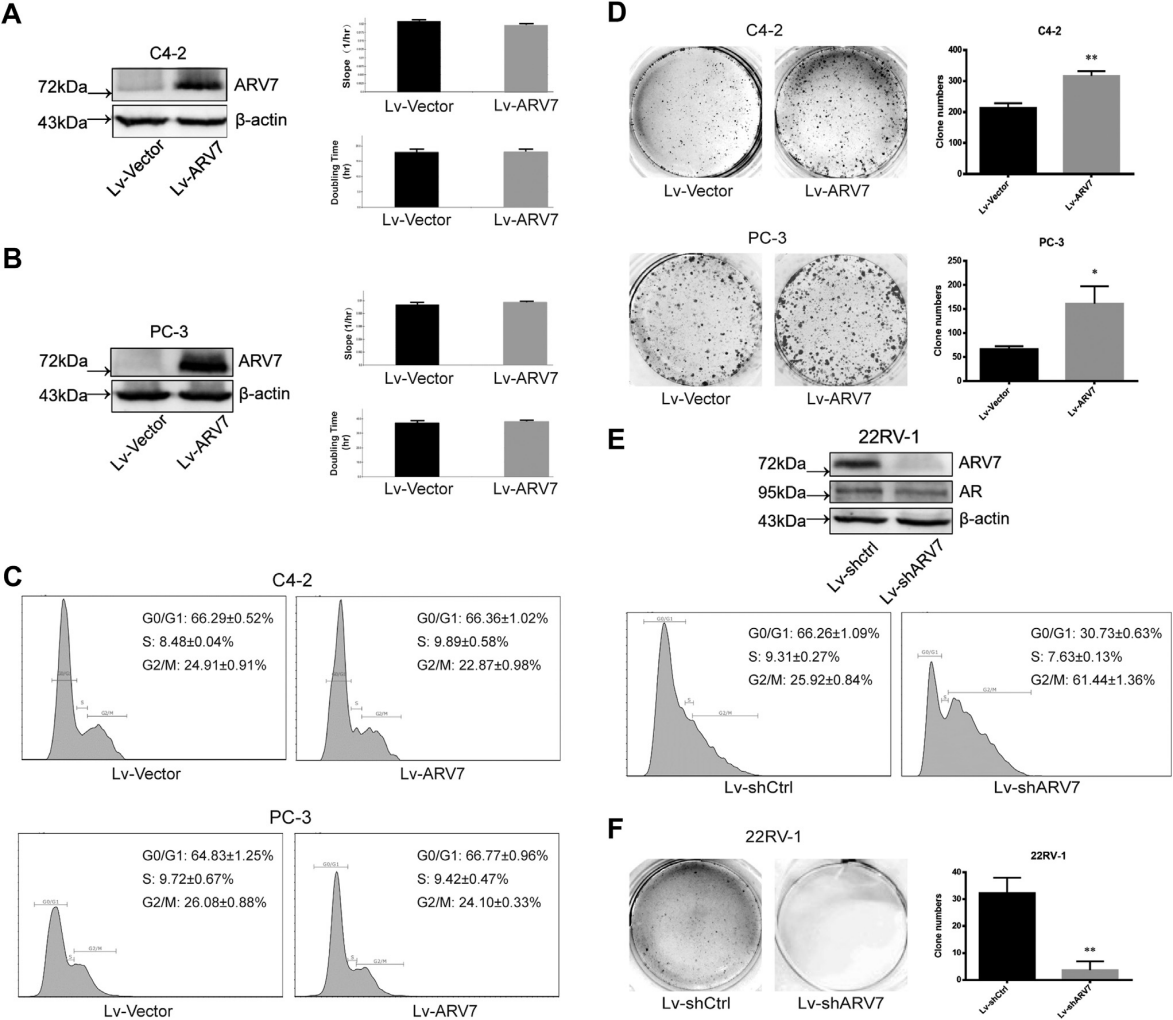
**ARV7 mediates oncogenic behaviors of PCa cells.***A*, stable virus-infected C4-2 cells (vector or ARV7 OE) were subjected to RTCA and immunoblotting (IB), respectively. Growth slope and doubling time were calculated by RTCA System and data were presented as mean ± SD, n = 3. *B*, stable virus-infected PC-3 cells (vector or ARV7 OE) were seeded for RTCA and IB. *C*, stable virus-infected C4-2 and PC-3 cells were subjected to FACS analysis, data were repeated at least three times, and percentages of each cell cycle stage were shown as mean ± SD. *D*, stable virus-infected C4-2 and PC-3 cells were seeded in 6-well plate (5000 cells/well for C4-2 and 1000 cells/well for PC-3) and cultured for 15 days, then cells were fixed and stained with 0.5% crystal violet, followed by quantification of at least three independent experiments, ∗*p* < 0.05, ∗∗*p* < 0.01. *E*, virus-infected 22RV-1 cells (shctrl or shARV7) were subjected to IB and FACS analysis. *F*, virus-infected 22RV-1 cells were seeded in 6-well plate (5000 cells/well) for colony formation assay.

First of all, we modified the expression level of ARV7 in several PCa cell lines to check whether ARV7 promotes oncogenic behaviors as we proposed. To our surprise, overexpression (OE) of ARV7 in C4-2 and PC-3 cells, both of which express undetectable level of endogenous ARV7, did not obviously benefit the growth of these cells (Fig. 1, *A* and *B*). In addition, we did not observe recognizable difference between ARV7 OE cells and control cells in cell cycle profile (Fig. 1*C*). However, in long-term culturing condition, ARV7-expressing cells showed significant stronger ability to form colonies than control group in both cell lines (Fig. 1*D*). Strikingly, depletion of endogenous ARV7 significantly impairs the growth of 22RV-1 cells, which normally express high level ARV7 (data not shown). Moreover, knockdown of ARV7 dramatically changes the cell cycle profile as more cells were arrested in G2/M phase (Fig. 1*E*). Consistent with those observations, ARV7 depletion almost completely abolished the colony formation ability of 22RV-1 cells (Fig. 1*F*). Taken together, these data indicate that ARV7 promotes the oncogenic behaviors of PCa cells and might play an important role in cell cycle progression.

### ARV7 level correlates with DTX sensitivity of PCa cells

**Figure 2:**
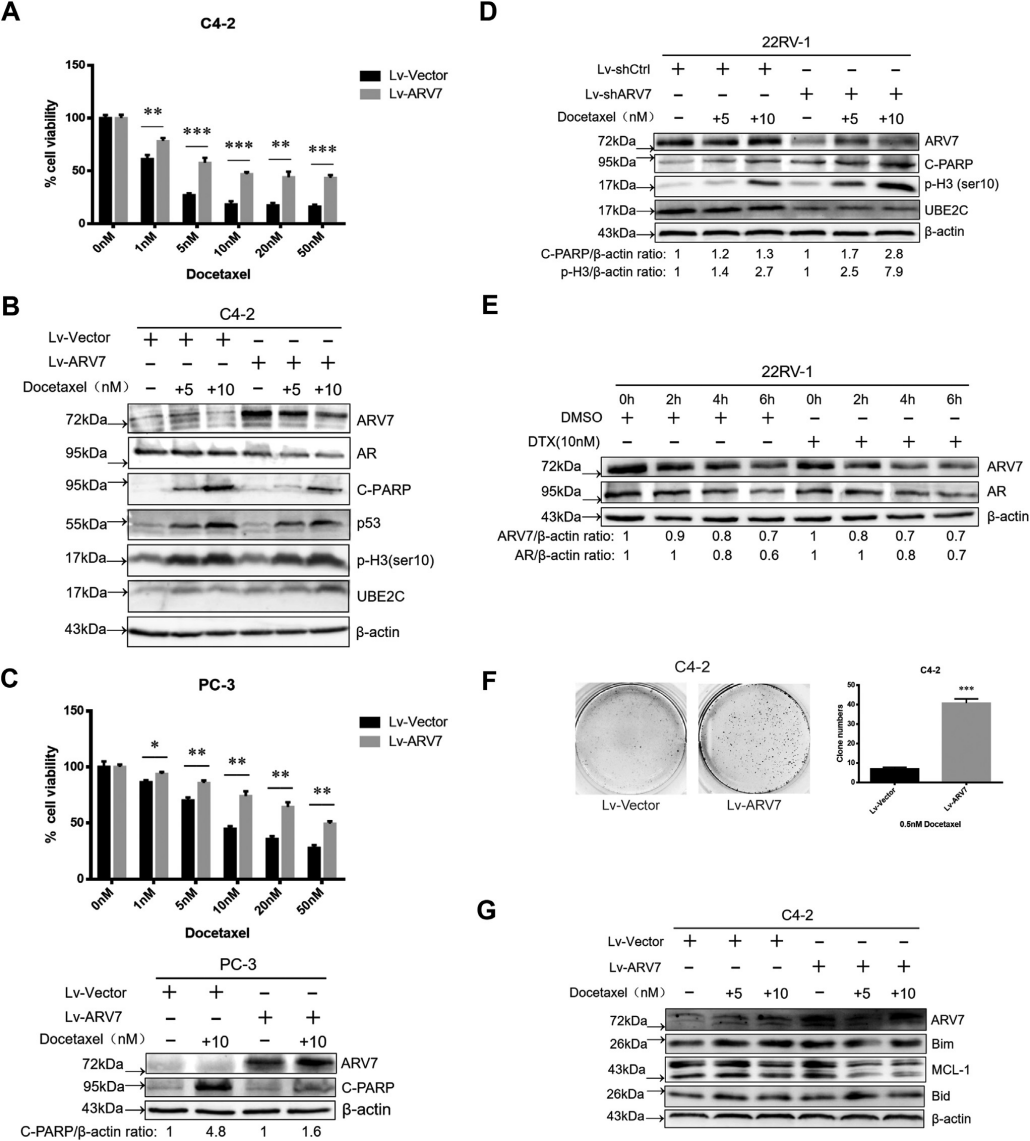
**ARV7 level correlates with DTX sensitivity of PCa cells.***A*, virus-infected C4-2 cells were seeded in 96-well plate, treated with DMSO or different concentration of DTX for 72 h, and then harvested for MTT assay, ∗∗∗*p* < 0.005. *B*, virus-infected C4-2 cells were treated with indicated concentration of DTX for 24 h and then harvested for IB. *C*, virus-infected PC-3 cells were treated with indicated concentration of DTX for 24 h and then reseeded in 6-well plate and 96-well plate for IB and MTT assay, respectively. *D*, virus-infected 22RV-1 cells were treated with indicated concentration of DTX for 24 h, and then cells were harvested for IB analysis. *E*, 22RV-1 cells were treated with DMSO or DTX for 24 h and then subjected to 100 μg/ml CHX treatment for indicated time before IB analysis. *F*, virus-infected C4-2 cells were seeded in 6-well plate (5000 cells/well), treated with 0.5 nM DTX for 15 days, medium was refreshed every 2 days, and then cells were fixed and stained, followed by quantification of the clones. *G*, virus infected C4-2 cells were treated with DMSO or DTX for 24 h and cell lysates were obtained for IB analysis.

Then, we used the methods above to investigate whether ARV7 level affects DTX sensitivity. Firstly, we found that C4-2 cells with stable ARV7 OE were more resistant to different concentrations of DTX treatments than cells infected with vector control (Fig. 2*A*). Moreover, as shown in Figure 2*B*, under DTX treatment, ARV7 OE cells induced much weaker level of apoptosis than control cells, which was indicated by less PARP cleavage. Consistently, similar results were obtained in PC-3 cells (Fig. 2*C*). On the other hand, after we depleted endogenous ARV7 in 22RV-1 cells, enhanced response to DTX treatment was observed (Fig. 2*D*). However, degradation of AR and ARV7 is not a major contributor to DTX cytotoxicity as we did not observe obvious change in the degradation rate of both AR and ARV7 after DTX treatment (Fig. 2*E*). Of note, under long time exposure to 0.5 nM DTX, ARV7-expressing C4-2 cells were able to form much more clones than control cells (Fig. 2*F*). Interestingly, as indicated in Figure 2*G*, upon modifying the level of ARV7, we did not detect obvious change of some apoptotic regulators, which were reported to be crucial for taxol-mediated mitotic death, including MCL-1 and BH3-only proteins, Bim and Bid (26, 27). In sum, these data indicate that ARV7 expression level is closely related to cellular sensitivity to DTX *in vitro*, but the intrinsic apoptosis regulators are not affected.

### ARV7 promotes mitotic slippage

**Figure 3:**
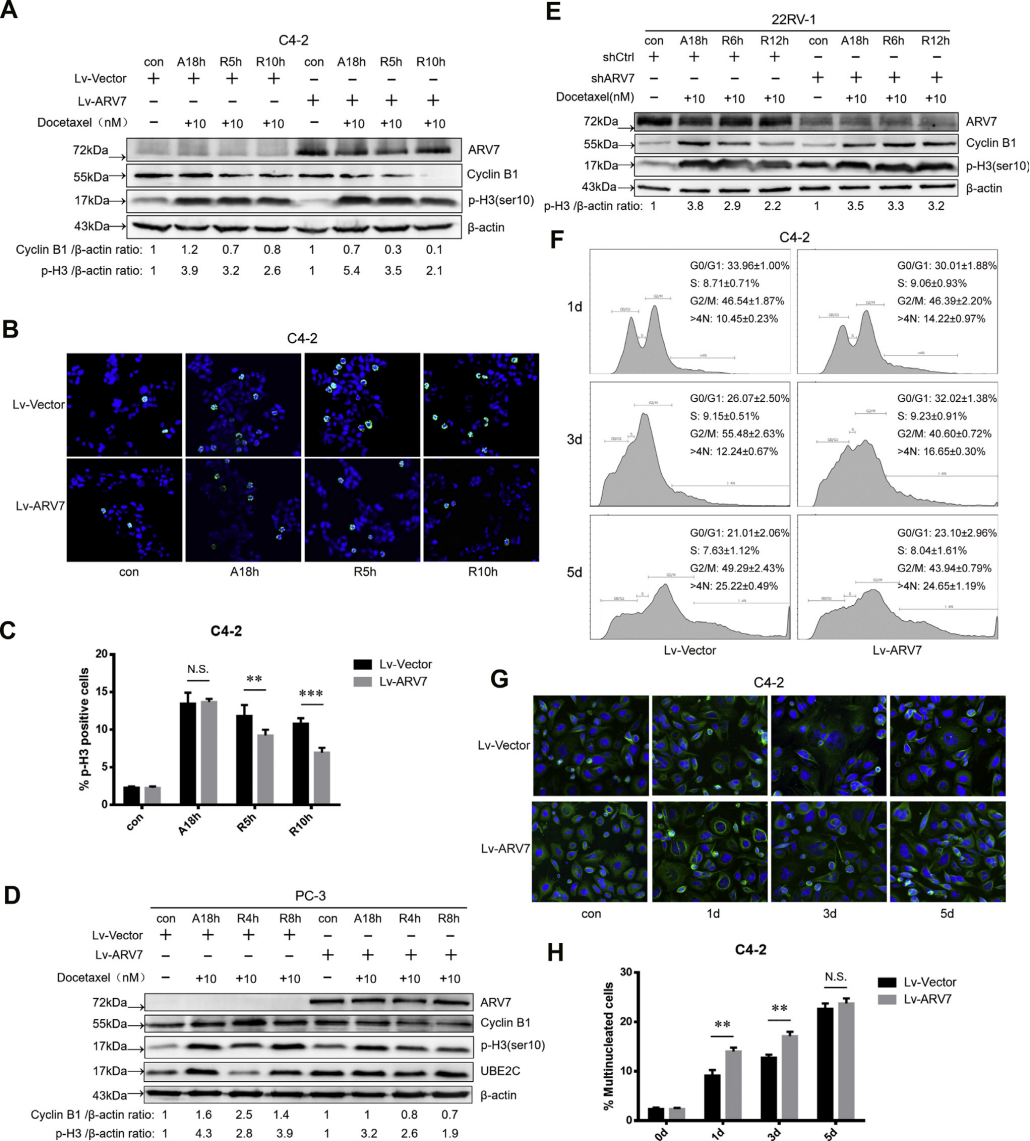
**ARV7 promotes mitotic slippage.***A*, virus-infected C4-2 cells were treated w/o 10 nM DTX for 18 h, then medium was refreshed to release the arrested cells, and cells were harvested at indicated time points, and finally, cell lysates were subjected to IB. *B*, virus-infected C4-2 cells were seeded on cover slips, cells were treated using the methods described in Figure 3*A* before subjected to immunofluorescence (IF) analysis, representative images of p-H3 positive cells(×20 fields) staining were showed for each group. *C*, quantification of p-H3 positive cells as percentage. For quantification, at least 100 cells were counted on each field (more than three sections at different groups). *D*, virus-infected PC-3 cells were treated with DTX arrest and release as previously described, and then cells were harvested for IB. *E*, virus-infected 22RV-1 cells (shctrl or shARV7) were treated with DTX arrest and release, and then cells were harvested for IB. *F*, virus-infected C4-2 cells were treated with 1 nM DTX for indicated times, and cells were harvested for FACS analysis, experiments were repeated at least three times, and percentages of each cell cycle stage were presented as mean ± SD. *G*, representative images of C4-2 cells treated with 1 nM DTX for different time, cells were stained for DAPI and alpha-tubulin for nucleus and cytoplasm, respectively. *H*, quantification of the multinucleated cells using the images acquired from Figure 3*G*, for each group, at least three fields were counted, data were presented as mean ± SD. Cells with giant nucleus(pseudo-G1 phase) were also scored as multinucleated cells.

Two independent networks compete with each other to determine the cell fate after microtubule poisons treatment, the mitotic death pathway slowly accumulates a death signal, and the mitotic slippage pathway slowly degrades cyclin B1, leading to mitotic exit. Since we didn’t detect obvious change of the intrinsic apoptotic pathway after modifying ARV7 expression level, we speculated that cells with ARV7 OE may have different cell cycle progression speed upon DTX treatment. Indeed, we found that after we released C4-2 cells from DTX treatment, cells with ARV7 OE showed increased degradation speed of cyclin B1 compared with control group (Fig. 3*A*). Also, the level of mitotic marker p-H3 (ser10) detected in ARV7 OE group dropped much faster and ARV7 OE group showed less p-H3 positive cells than control group, indicating that ARV7 OE group can exit DTX-induced mitotic arrest more quickly (Fig. 3, *B* and *C*). Consistently, we also observed similar results in PC-3 cells (Fig. 3*D*). On the other hand, DTX arrested cells showed slower speed to exit mitosis when we depleted endogenous ARV7 in 22RV-1 cells (Fig. 3*E*). Because cells undergoing mitotic slippage can exit mitosis without cytokinesis and subsequently generate multinucleated cells, we next designed experiments to check whether ARV7 regulates this process after DTX treatment. As the data shown in Figure 3*F*, after different time exposure to low concentration of DTX, ARV7 OE group contained more cells with DNA content more than 4N and even DNA content with 8N, representing aneuploid and polyploid cells, respectively. To further confirm this observation, we also quantified the percentage of multinucleated cells using immunofluorescence images. The data showed that under low-dosage DTX treatment, ARV7 OE group displayed more multinucleated cells than control group (Fig. 3, *G* and *H*). Collectively, those data indicate that ARV7 promotes mitotic slippage under DTX treatment.

### The SAC activity is impaired in ARV7 expressing cells

**Figure 4:**
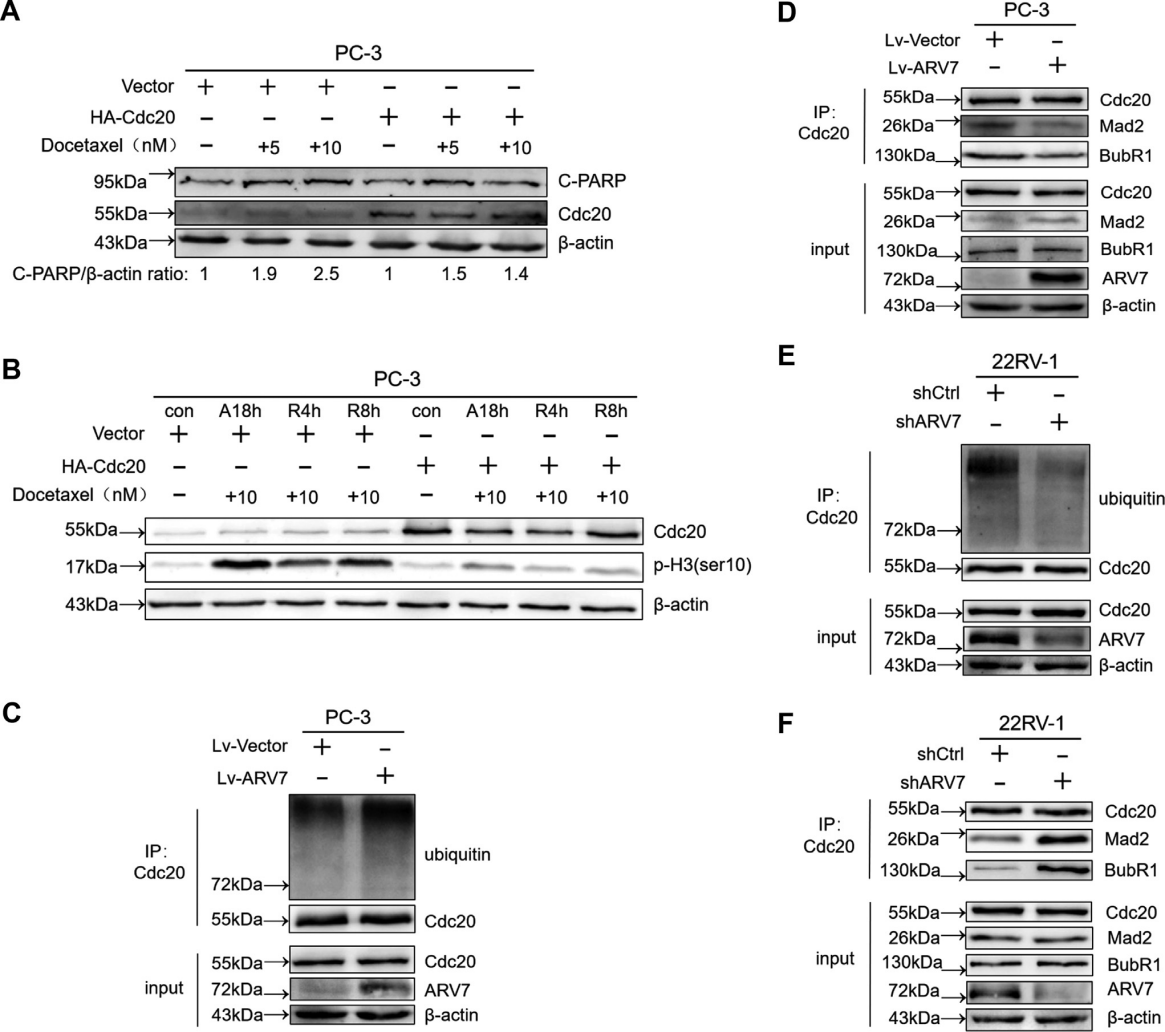
**ARV7 inhibits the SAC function.***A*, PC-3 Cells were transfected with vector control or HA-CDC20 for 24 h and then reseeded in 6-well plate, treated with indicated concentration of DTX for another 24 h before harvesting for IB. *B*, transfected PC-3 cells were reseeded in 6-well plate and subjected to DTX arrest and release treatment, cells were harvested at different time points as indicated, and then IB experiments were performed using the cell lysates. *C*, virus-infected PC-3 cells were arrested using 10 nM DTX for 18 h, then medium was refreshed to release the cells with the presence of 10 μM MG132, after 8 h, cells were harvested for IB and immunoprecipitation (IP). *D*, virus-infected PC-3 cells were arrested using 10 nM DTX for 18 h, then medium was refreshed to release the cells for additional 8 h before harvesting for IB and IP. *E*, virus-infected 22RV-1 cells (shCtrl or shARV7) were treated as 4(C) described and subjected to IP and IB. *F*, virus-infected 22RV-1 cells were treated as 4(D) described and subjected to IP and IB.

In order to induce slippage from DTX-induced mitotic arrest, the MCC must be disassembled and the coactivator Cdc20 has to be released to enable reactivation of the APC/C. Indeed, when we overexpressed Cdc20 to increase free Cdc20 level in the cells, cells were more resistant to DTX treatment and could exit from DTX-induced mitotic arrest more easily (Fig. 4, *A* and *B*). Previous study has identified that Cdc20 needs to be multiubiquitinated by the APC/C before releasing from the MCC. Hence, we checked the ubiquitination level of Cdc20 upon modifying the ARV7 expression level in PCa cells. As shown in Figure 4*C*, ubiquitination level of Cdc20 was significantly higher in ARV7-expressing cells after DTX release. Moreover, the bindings between Cdc20 and two crucial MCC components, including Mad2 and BubR1, were impaired in ARV7 OE cells, suggesting that ARV7 inhibits the stability of the MCC (Fig. 4*D*). In addition, when we depleted endogenous ARV7 in 22RV-1 cells, the poly-ubiquitination level of cdc20 was reduced while the bindings between cdc20 and the MCC components were enhanced during DTX arrest and release treatment (Fig. 4, *E* and *F*). Taken together, these results above suggest that ARV7 inactivates the SAC through promoting the disassembly of Cdc20 from the MCC during DTX treatment.

### UBE2C regulates mitotic slippage and DTX sensitivity

**Figure 5:**
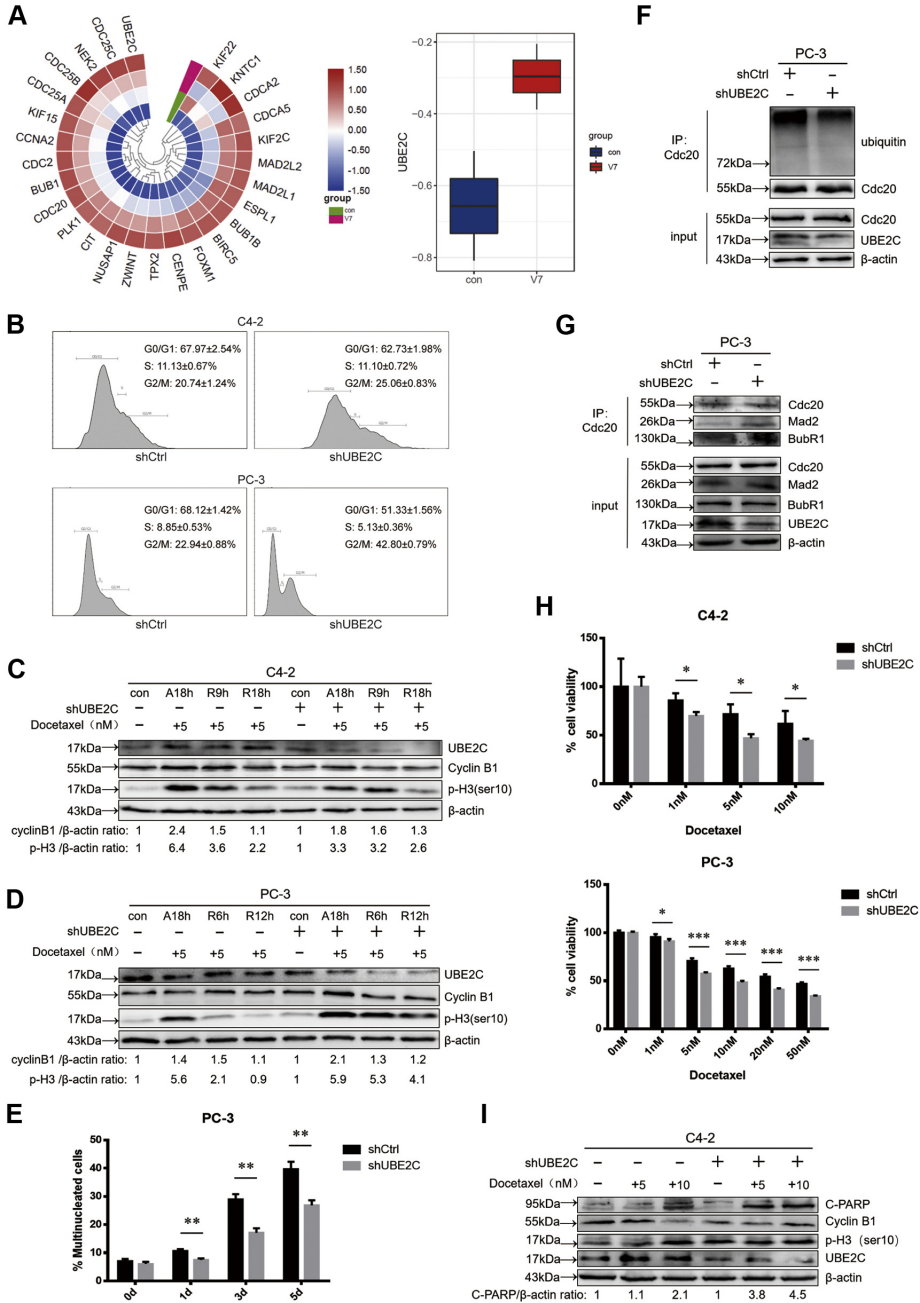
**UBE2C mediates DTX sensitivity and mitotic slippage of PCa cells.***A*, heatmap representing the expression profiles of several mitotic genes associated with ARV7 status using the public database, specifically, the correlation between ARV7 and UBE2C was shown as the box plot. *B*, virus-infected C4-2 and PC-3 cells (vector or shUBE2C) were harvested for FACS analysis. *C* and *D*, virus-infected C4-2 cells or PC-3 cells were treated with DTX arrest and release as previously described, after indicated time, cells were harvested for IB. *E*, quantification of the multinucleated cells using the IF images acquired. *F*, virus-infected PC-3 cells were arrested using 10 nM DTX for 18 h, then medium was refreshed to release the cells with the presence of 10 μM MG132, after 8 h, cells were harvested for IB and IP. *G*, virus-infected PC-3 cells were arrested using 10 nM DTX for 18 h, then medium was refreshed to release the cells for additional 8 h before harvesting for IB and IP. *H*, virus-infected C4-2 and PC-3 cells were seeded in 96-well plate, treated with DTX or DMSO for 72 h before harvesting for MTT assay. *I*, virus-infected C4-2 cells were treated with indicated concentration of DTX for 24 h and then harvested for IB.

Next, we move on to investigate how ARV7 regulates mitotic slippage and the SAC activity by checking some of its potential targets. On the basis of previous analysis, we found that ARV7 expression is positively correlated with many genes involved in mitotic progression, microtubule dynamic, and kinetochore functions, all of which are closely related to the cellular effect of DTX. Among those targets with high enrichment scores, *UBE2C* is one of the cell-cycle-related *genes* considered to be actively involved in mitotic exit (Fig. 5*A*). Of note, UBE2C is also demonstrated to be the E2 enzyme required for the APC/C to degrade cyclin B1 and also crucial for the SAC inactivation (28, 29). Based on these findings, we believe that UBE2C might be the core downstream factor of ARV7 to regulate the mitotic slippage pathway. Firstly, consistent with the role of UBE2C in mitotic exit, we found that upon UBE2C depletion, cells showed increased mitotic index (Fig. 5*B*). More importantly, UBE2C-depleted cells showed slower degradation rate of cyclin B1 and p-H3 (ser10) after releasing from DTX treatment, indicating that it is more difficult for cells with UBE2C silencing to recover from DTX-induced mitotic arrest (Fig. 5, *C* and *D*). In addition, much less multinucleated cells were observed in shUBE2C group than control group under low-dosage DTX treatment (Fig. 5, *E*, images not shown). These data strongly suggest that UBE2C plays a crucial role in mitotic slippage. In support of these observations, we also found that after UBE2C depletion, the ubiquitination level of Cdc20 after DTX release was decreased while the bindings between Cdc20 and Mad2/BubR1 were enhanced (Fig. 5, *F* and *G*). Next, we checked whether UBE2C affects cellular sensitivity to DTX. As Figure 5, *H* and *I* shows, UBE2C depleted cells were more sensitive to DTX treatment than control cells and DTX tended to induce stronger apoptosis level in shUBE2C group. In sum, these data demonstrate that UBE2C is a crucial molecule responsible for regulating mitotic slippage and DTX sensitivity.

### ARV7 mediates effect of DTX through mitotic slippage

**Figure 6:**
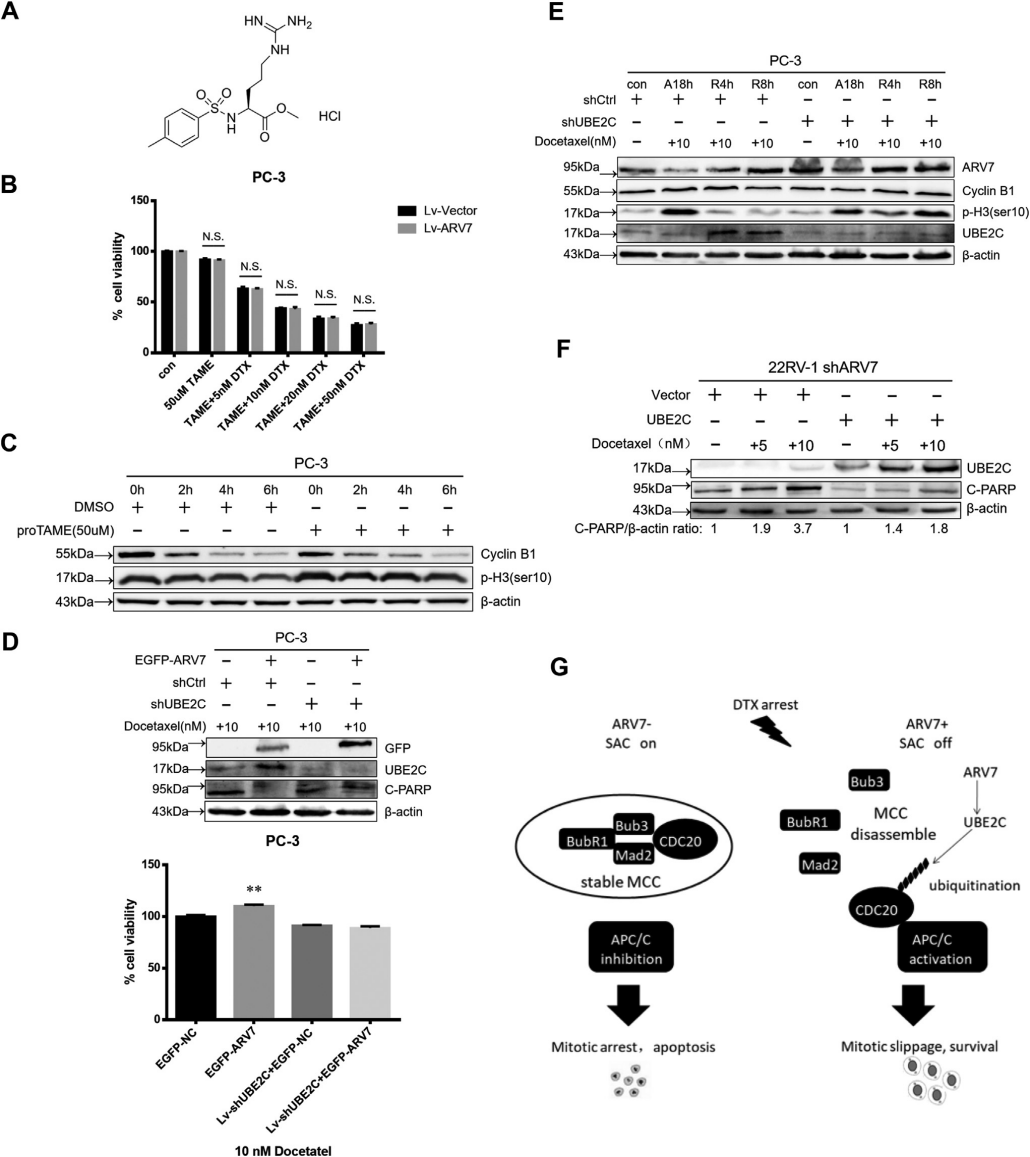
**ARV7 mediates DTX sensitivity through the mitotic slippage pathway.***A*, chemical structure of the APC/C inhibitor proTAME. *B*, virus-infected PC-3 cells were cotreated with 50 μM proTAME and different concentrations of DTX for 72 h, and then cells were harvested for MTT assay. *C*, PC-3 cells were treated with DTX arrest for 18 h, then medium was refreshed to release the cells in the presence of 100 μg/ml CHX (DMSO/50 μM proTAME was kept in the medium for the entire experiments), after indicated time points, cells were harvested for IB. *D*, virus-infected PC-3 cells (vector or shUBE2C) were transfected with indicated plasmid for 24 h and reseeded for IB and MTT assay after DTX treatment. *E*, virus-infected PC-3 cells (vector or shUBE2C) were transfected with EGFP-ARV7 plasmid and subjected to DTX arrest and release treatment before harvesting for IB. *F*, ARV7-depleted 22RV-1 cells were transfected with vector control or UBE2C for 24 h and then treated with or without indicated concentration of DTX for another 24 h before IB analysis. *G*, proposed model for the study, ARV7 status affects the function of the SAC, subsequently regulating the cell fate after DTX treatment.

To further confirm that ARV7 mediates DTX sensitivity through the mitotic slippage pathway and UBE2C, we performed the following experiments. Firstly, we found that if we cotreated cells with the APC/C specific inhibitor proTAME, OE of ARV7 cannot promote survival of PCa cells under the treatment of DTX as efficient as previously shown (Fig. 6, *A* and *B*). Since proTAME can efficiently inhibit mitotic exit, ARV7 might at least partially lose its beneficial effect on survival during the mitotic arrest condition (Fig. 6*C*). Importantly, as illustrated in Figure 6, *D* and *E*, when we depleted UBE2C under the condition of ARV7 OE, ARV7 not only loses its ability to significantly increase cell survival but also fails to efficiently promote premature mitotic exit after DTX treatment. Furthermore, ARV7-depleted 22RV-1 cells can regain their resistance to DTX treatment after we overexpressed UBE2C using plasmid (Fig. 6*F*). Overall, these data above support our proposed model that ARV7 mediates DTX sensitivity through the mitotic exit and UBE2C regulation (Fig. 6*G*).

### Inhibiting mitotic slippage enhances the toxicity of DTX in ARV7-expressing PCa cells

**Figure 7:**
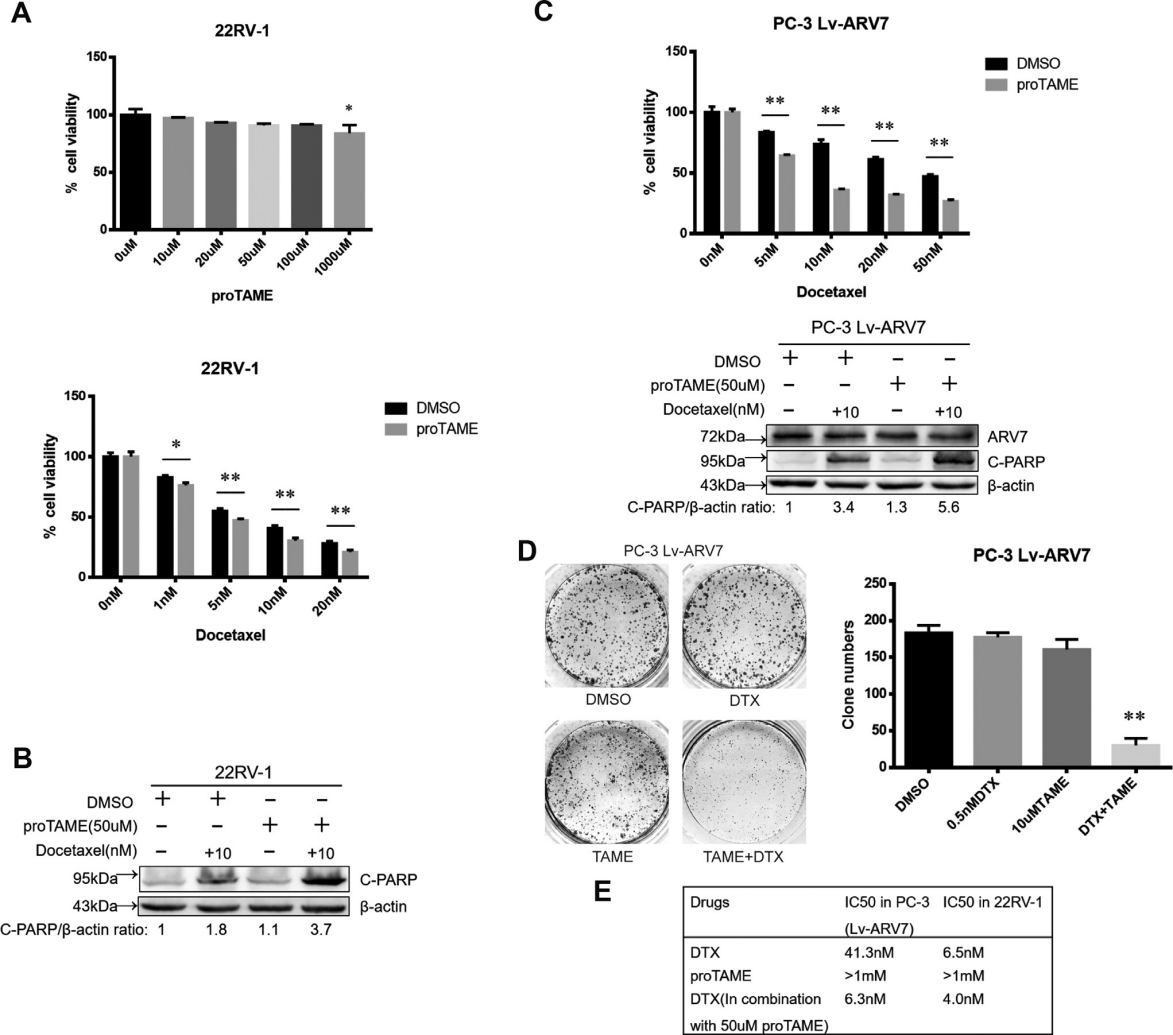
**Inhibiting mitotic slippage enhances the toxicity of DTX in ARV7-expressing PCa cells.***A*, *Upper panel*, 22RV-1 cells were subjected to different concentration of proTAME for 72 h and harvested for MTT assay, ∗*p* < 0.05 compared with the DMSO control. *Lower panel*, 22RV-1 cells were treated with different concentration of DTX alone or in combination with 50 μM proTAME for 72 h and then harvested for MTT assay. *B*, 22RV-1 cells were treated with proTAME, DTX, or proTAME plus DTX for 24 h and then subjected to IB. *C*, *Upper panel*, virus-infected PC-3 cells were subjected to different drug treatments as indicated and harvested for MTT assay. Lower panel, virus-infected PC-3 cells were treated with proTAME, DTX, or proTAME plus DTX for 24 h and then subjected to IB. *D*, virus-infected PC-3 cells were seeded in 6-well plate (1000 cells per well) treated with (DMSO as control) 0.5 nM DTX, 10 μM proTAME, or both for 15 days, medium was refreshed every 2 days, and then cells were fixed and stained, followed by quantification of the clones. *E*, IC50 values of DTX and proTAME in 22RV-1 and ARV7-expressing PC-3 cells.

Based on these observations, we speculated that combination of the APC/C inhibitor and DTX will have a synergistic effect in ARV7-expressing PCa cells. As a result, although single treatment with high-concentration proTAME had little effect on the survival of 22RV-1 cells, cotreatment with proTAME significantly increased the cytotoxicity of DTX, suggesting a strong synergistic effect of these two drugs in ARV7-expressing PCa cells (Fig. 7*A*). In addition, combination treatment of 22RV-1 cells with DTX and proTAME induced much stronger level of apoptosis than single drug treatment (Fig. 7*B*). Consistently, we also observed the similar results using ARV7-expressing PC-3 cells (Fig. 7*C*). Finally, under longer treatment time, DTX and proTAME act synergistically to inhibit colony formation of ARV7-expressing PC-3 cells (Fig. 7*D*). Thus, although we cannot calculate the combination index for these two drugs due to the high IC50 of proTAME in the PCa cell lines, we were able to observe the strong synergy in inhibiting the viability of ARV7-expressing PCa cells (Fig. 7*E*).

## Discussion

Although DTX has been long served as the first-line chemotherapeutic agent for mCRPC, precisely how it exerts cytotoxic effects is perplexing. This is largely due to the fate of cells after prolonged mitotic arrest varies dramatically in different cell lines and even among individual cells from the same line. Recently, researchers believe that the cell fate after DTX treatment appears to be determined by two independent and competing networks, one controlling mitotic cell death and the other mediating mitotic slippage. Mitotic slippage occurs in the presence of the SAC signaling, which is crucial for cells to escape from DTX-induced mitotic arrest (26). However, how the MCC stability and the APC/C activity are actively controlled during PCa progression remains largely unknown. Based on previous findings, ARV7 not only serves as the major contributing factor for CRPC progression but also demonstrated to promote DTX resistance even the detailed mechanisms are lacking. The notion that unlike its parental AR, ARV7 might regulate a set of cell-cycle-related genes motivates us to investigate the unique role of ARV7 in cell cycle regulation (Fig. 5*A*). Strikingly, we found that depletion of ARV7 in 22RV-1 cells dramatically induced mitotic arrest (Fig. 1*E*). Because mitotic progression and the SAC activity are vital for DTX-induced cell death, finding the potential relationship between ARV7 and these processes is of great significance in order to understand the role of ARV7 in DTX resistance.

In this study, we are the first group to show that ARV7 mediates DTX sensitivity through inactivating the SAC and promoting mitotic slippage rather than affecting the apoptotic regulators. Consistent with our findings, previous experiments have shown that taxol-resistant cells have impaired activity of the SAC and enhanced expression of ARV7 (22, 30). Furthermore, we also identified UBE2C as the downstream effector of ARV7 to regulate the MCC disassociation and the APC/C reactivation. Interestingly, UBE2C was initially identified as a target of AR-FL during androgen-independent state (9). In addition, some of the ARV-mediated oncogenic functions were shown to be dependent on AR-FL, suggesting that ARV expression serves as an adaptive reaction to hormone therapy and the interplay between AR-FL and ARV might play a crucial role in mediating those processes (31). However, following studies have claimed that ARV7 is the major driver for those mitotic genes and the promoter of UBE2C was bound by AR-V7 only (32, 33). Thus, it is necessary to further investigate the regulatory mechanism of UBE2C expression under different AR-FL/ARV7 context in future experiments. Of note, some earlier experiments have demonstrated that cells overexpressing UBE2C ignore the mitotic spindle checkpoint signals and lose genomic stability, which contributes to cancer progression (34, 35). Considering that UBE2C plays a crucial role in the APC/C activation, and Cdc20 was also indicated to be regulated by ARV7 in earlier reports, it is important to further investigate the role of ARV7 in the network of mitotic exit regulation (36, 37). In addition, as our data supported that the APC/C inhibitor can efficiently enhance the cytotoxicity of DTX in ARV7-expressing CPRC cells, it is possible to offer a novel path to combat against taxol resistance of mCRPC patients, especially those with high ARV7 expression profiles.

In general, our results showed that ARV7 promotes DTX resistance largely by promoting the SAC silencing and mitotic slippage as the mitotic death is the major determinant for DTX toxicity at least in short-term, cell culture conditions. However, considering the *in vivo* situations, DTX cannot induce mitotic arrest as strong as it does in cell culture due to concentration and pharmacokinetic issues. In other words, the effect of mitotic death in clinics has been significantly overwhelmed by slippage-associated events. Thus, that is probably the reason why some earlier clinical assessments failed to connect ARV7 status to the DTX response of patients as the cellular assay claimed, leading to the debate whether ARV7 actually relates to DTX efficacy (23, 38, 39). It is postulated that the real efficacy of mitotic poisons in clinical therapy is determined by the chromosome defects-induced DNA damage and the inflammation or immunological factors associated with those polyploid cells under chronic, low-dosage treatment (26, 40, 41). Postslippage cells can either undergo apoptosis as the consequence of intense DNA damage or enter senescence. Remarkably, those senescent cells are capable of metabolizing some factors closely related to tumor microenvironment and inflammation, which is termed as senescence-associated secretary phenotype (SASP) (17, 42). Thus, as we are almost completely blind about how ARV7 associated with those pathways, it is still too preliminary to state that ARV7 is a biomarker for DTX therapeutic response. Nevertheless, based on the novel findings about the regulation of mitotic slippage, we could gain inspiration to further assess the functions of ARV7 in those postslippage cells in future, searching for better and more specific targets for overcoming DTX resistance.

## Experimental procedures

### Chemicals

DTX and puromycin powder were purchased from MedChemExpress while G418, MG132, and CHX were purchased from Sigma. The APC/C inhibitor proTAME was purchased from Merck Millipore and dissolved in DMSO.

### Cell culture and plasmid transfection

PC-3, C4-2, and 22RV-1 cells were originally purchased from ATCC, and LNCaP cells were kindly provided by StemCell Bank, Chinese Academy of Sciences. LNCaP, C4-2, and 22RV-1 cells were cultured in Roswell Park Memorial Institute 1640 medium (Gibco Life Technologies) supplemented with 10% fetal bovine serum, 100 U/ml penicillin, and 100 U/ml streptomycin in 5% CO2 at 37 °C while PC-3 cells were cultured in Dulbecco's modified Eagle medium/F-12 (Gibco) medium using the same condition. For plasmid DNA transfection into cells, either TurboFect Transfection Reagent (Thermo Fisher Scientific) or Lipofectamine 2000 (Invitrogen) was used according to the manufacturer’s recommended protocols. The EGFP-ARV7 plasmid was purchased from Addgene while HA-Cdc20 was purchased from GenePharm.

### Lentivirus infection

For generating cells stably expressing ARV7 or cells with ARV7 depletion, lentiviral particles were synthesized by GenePharm, and infected cells were selected using puromycin as the protocol recommended. The sequence for ARV7 expression is in accordance with NCBI database (NM_001348061.1) while the targeting sequence for ARV7 knockdown is 5’-GCTCCATAGCTTCCATATTGA-3’ within the 3’-UTR region. For UBE2C depletion, human UBE2C shRNA lentiviral particles (sc-61742-v) were purchased from Santa Cruz Biotechnology, and UBE2C depleted cells were selected using puromycin as well.

### Immunoblotting and immunoprecipitation

Cell lysates were prepared using RIPA Buffer (Boston BioProducts) supplemented with protease inhibitors (Sigma) and phosphatase inhibitor (Active Motif). For immuoprecipitation experiments, cell lysates were incubated with desired antibodies in Tris buffered saline buffer at 4 °C overnight, then protein G agarose (Beyotime Biotechnology) was added to each sample for another 2 h incubation, followed by five times wash with PBS solution and boiling for IB. To avoid the interference from denatured IgG heavy chain, VeriBlot IP Detection Reagent (ab131366, Abcam) was used for IP detection. The antibodies against ARV7 (ab198394), Bcl-2 (ab59348), MCL-1 (ab28147) and Bim (ab15184) were obtained from Abcam while antibodies against cleaved-PARP (5625), Bid (2002), UBE2C (14234), p-H3(ser10) (9701), BubR1 (4116), and Cdc20 (14866) were purchased from Cell Signaling Technology, and the antibodies against GFP (sc-9996), p53 (sc-126), Mad-2 (sc-47747), and ubiquitin (sc-8017) were obtained from Santa Cruz. Proteintech is the provider of antibodies against β-actin.

### Immunofluorescence staining

Cells were washed and fixed in 4% (w/v) paraformaldehyde/PBS for 20 min and subjected to proteinase K digestion for 1 min and then permeabilized with 0.1% Triton X-100 for 15 min. After blocking with bovine serum albumen (BSA) for 30 min, cells were incubated with primary antibody overnight at 4 °C. The next day, cells were stained with FITC/Texas Red-conjugated secondary antibodies (1:200 dilution, Proteintech) for 30 min in the dark. Finally, cells were treated with DAPI for 2 min, and images were acquired by an Olympus FV1000 confocal laser microscope.

### Flow cytometry

Cells were seeded in 6-well plates. After exposure to different treatment, cells were collected and PI staining was performed according to the manufacturer's instructions. Then, samples were analyzed by Accuri C6 Flow Cytometry (BD Biosciences).

### Cell viability assay

Virus-infected or plasmid-transfected cells were seeded in 96-well plates, treated w/o indicated drugs or DMSO as control for 72 h. Then, media was removed and 0.5 mg/ml MTT was added for 4 h. Formazan crystals were dissolved with 150 μl DMSO and absorbance was measured at a wavelength of 490 nm.

### Real-time cell analysis (RTCA)

The RTCA S16 System (ACEA Biosciences) was used to monitor cellular status. Briefly, cells were seeded into a special 16-well electronic plate (16-E-Plate). Then, the plate was placed into the special station and connected to an electronic sensor analyzer by electrical cables. Then the station was placed in the culturing CO2 incubator. The more the cells there are on the electrodes, the larger the change in electrode impedance. A unitless parameter termed Cell Index is used to measure the relative change in electrical impedance to represent cell status. The accompanying software was used to carry out the dimensionless impedance-based Cell Index. The Cell Index value changes with time and reflects the number of cells inside the well.

### Colony formation assay

After cells (with desired virus infection or drug treatment) were seeded in 6-well plates (1000–5000 cells/well) for 15 days with medium refreshment every 2 days, cells were fixed with 10% formalin and stained with 0.05% crystal violet.

### Microarray analysis

The raw data of mRNA expressions in prostate cancer cells were downloaded from GEO (https://www.ncbi.nlm.nih.gov/geo/) (GSE36549). We filtered to include only the samples overexpressing ARV7 and its control group. Processing of gene expression data was carried out using the Limma package in the R software. Data in each array was normalized using quantile normalization procedure.

### Statistical analyses

The level of significance indicated by *p*-values was calculated using standard two-sided Student’s *t*-tests. *p* < 0.05 was considered statistically significant.

## Data availability

The majority of data that support the findings of this study are included within the article and the remaining results, which are not displayed in the article, are available from the corresponding author upon reasonable request.

## Conflict of interest

The authors declare no potential conflicts of interest.
